# Influenza A Virus and Acetylation: The Picture Is Becoming Clearer

**DOI:** 10.3390/v16010131

**Published:** 2024-01-17

**Authors:** Matloob Husain

**Affiliations:** Department of Microbiology and Immunology, University of Otago, P.O. Box 56, Dunedin 9054, New Zealand; matloob.husain@otago.ac.nz

**Keywords:** influenza virus, acetylation, HDACs, HATs, epigenetic modification, protein modification, deacetylases, acetyltransferases, IAV

## Abstract

Influenza A virus (IAV) is one of the most circulated human pathogens, and influenza disease, commonly known as the flu, remains one of the most recurring and prevalent infectious human diseases globally. IAV continues to challenge existing vaccines and antiviral drugs via its ability to evolve constantly. It is critical to identify the molecular determinants of IAV pathogenesis to understand the basis of flu severity in different populations and design improved antiviral strategies. In recent years, acetylation has been identified as one of the determinants of IAV pathogenesis. Acetylation was originally discovered as an epigenetic protein modification of histones. But, it is now known to be one of the ubiquitous protein modifications of both histones and non-histone proteins and a determinant of proteome complexity. Since our first observation in 2007, significant progress has been made in understanding the role of acetylation during IAV infection. Now, it is becoming clearer that acetylation plays a pro-IAV function via at least three mechanisms: (1) by reducing the host’s sensing of IAV infection, (2) by dampening the host’s innate antiviral response against IAV, and (3) by aiding the stability and function of viral and host proteins during IAV infection. In turn, IAV antagonizes the host deacetylases, which erase acetylation, to facilitate its replication. This review provides an overview of the research progress made on this subject so far and outlines research prospects for the significance of IAV-acetylation interplay.

## 1. Influenza A Virus

Influenza virus causes an acute febrile respiratory disease, influenza, in humans that is commonly known as the “flu”. Influenza virus is one of the most circulated human viruses, and the flu is one of the most prevalent infectious human diseases globally. Influenza virus is endemic in humans and causes recurring seasonal flu epidemics in the winter months in temperate climates and the rainy season in tropical climates [1]. Nevertheless, influenza virus remains active throughout the year in the global human population due to alternate winter seasons in the Northern and Southern hemispheres. These flu epidemics account for an estimated one billion cases, which include 3–5 million severe cases needing hospitalization and 290,000–650,000 deaths annually [2]. In addition, influenza virus also causes occasional pandemics and sporadic zoonotic outbreaks. The former can cause several billions of cases within a few months, whereas the latter cause few cases but with higher mortality rates (30–50%) [3]. In the future, the co-infections of influenza virus with SARS-CoV-2 may impose an additional burden on global public health [4]. Influenza virus belongs to the *Orthomyxoviridae* family and exists in four types: A, B, C, and D [5].

Influenza A virus (IAV) is the most significant among the four types, because it is the only influenza virus which is known to cause all three flu events—recurring seasonal epidemics, unpredictable pandemics, and deadly zoonotic outbreaks [6,7]. IAV has been part of the ecosystem for centuries and will remain so for the foreseeable future. There are three main reasons for this prediction: (1) IAV has a broad host range, (2) IAV possesses an RNA genome, and (3) IAV’s RNA genome has a segmented configuration [5]. IAV infects a variety of mammalian and avian hosts that inhabit all three main media of the environment: (1) water (e.g., seals and waterfowl), (2) land (e.g., humans, pigs, chickens, cats, and horses), and (3) air (e.g., migratory birds, and bats). Such a repertoire of host species creates opportunities for IAV to undergo regular inter-species transmission and constantly circulate in the environment [7]. IAV is an enveloped virus with a segmented, single-stranded, negative-sense RNA genome [5]. The IAV particles can be spherical and filamentous in shape with a diameter of ~100 nm and a length of ~20 µm, respectively [5]. IAV particles are composed of a host-derived lipid envelope that incorporates viral membrane proteins, hemagglutinin (HA), neuraminidase (NA), and matrix protein 2 (M2). Underneath the envelope, viral matrix protein 1 (M1) forms a supporting layer, and in the centre lie eight viral ribonucleoprotein (vRNP) complexes. Each vRNP contains a viral RNA segment, viral nucleoprotein (NP), and three viral RNA-dependent RNA polymerase subunits—polymerase acidic (PA), polymerase basic 1 (PB1) and polymerase basic 2 (PB2). For infection, IAV attaches to the host cells by binding to the sialic acid receptors via HA and enters the cells via receptor-mediated endocytosis. The vRNPs are released into the cytoplasm and trafficked to the nucleus for viral RNA transcription and replication. Notably, IAV is one of the few RNA viruses which replicate in the nucleus [5]. After the synthesis of all viral components and the initial assembly of vRNPs in the nucleus, all the components are trafficked to the host cell plasma membrane for final vial progeny assembly and release by budding; the latter occurs via the action of NA [5]. 

The IAV RNA-dependent RNA polymerase is an error-prone enzyme and trades efficiency for accuracy when making copies of the RNA genome for millions of IAV offspring in a single infection cycle. This results in continuous genetic drift with ~1 substitution occurring per 5000 nucleotides. Consequently, each viral progeny ends up with at least one mutation [8,9]. Such genetic drift, if occurring in the genes of IAV surface antigens HA and/or NA, leads to antigenic drift. These drifted IAV variants can evade the adaptive immunity generated by previous IAV infections or vaccinations and cause seasonal flu epidemics, hence the need for annual flu vaccinations [10]. The segmented nature of the IAV genome creates opportunities for an IAV subtype to exchange gene segments with another IAV subtype if two or more IAV subtypes infect the same host, e.g., pig, and replicate in the same host cell. This phenomenon is known as genetic shift or gene reassortment, and like antigenic drift, if this occurs with the HA and/or NA genes, it becomes antigenic shift. If the resulting IAV variants then jump to and establish in a human population, it can lead to a flu pandemic [10]. So far, IAV has been recorded to have caused four flu pandemics, which occurred in 1918, 1957, 1968 and 2009. The 2009 pandemic was caused by an IAV variant that originated in pigs [7]. Recent outbreaks, spread, evolution, and transmission of avian IAVs, particularly of the resurgent H5N1 subtype in various animals and continents, make them a potential pandemic threat [11,12,13,14,15,16,17,18,19,20,21]. Therefore, it is a matter of when, not if, the next flu pandemic will occur. A universal influenza vaccine has yet to be developed but the influenza virus type- and subtype-specific vaccines are available. Furthermore, three classes of anti-influenza virus drugs, M2, NA, and PA inhibitors, are also available [22]. However, the constantly evolving nature of the influenza virus, via genetic drifts and shifts, challenges vaccine efficacy [10] and allows drug resistance to emerge [22]. Therefore, it is critical to continue studying IAV to design effective antiviral interventions.

## 2. Influenza A Virus Pathogenesis and Proteome Complexity

IAV infects humans of all ages and ethnicities and targets the human airways to cause the flu. However, the flu can be severe in children aged 5 and below, adults aged 65 and above [23], and some ethnic populations [24]. The symptoms and severity of the flu can vary in humans, ranging from upper respiratory tract illnesses like tracheobronchitis, pharyngitis, fever, and myalgia to lower respiratory tract illnesses like pneumonia and acute respiratory distress syndrome (ARDS) [23,25]. Several viral proteins and host proteins, as well as the host conditions, e.g., age, immune status, and chronic diseases, determine the severity and duration of IAV infection and disease symptoms. IAV possesses eight genes (HA, M, NA, NP, NS, PA, PB1, and PB2), each of which encodes one protein to several proteins [5]. The latter occurs via alternative splicing, ribosomal frameshifting, or alternate start codon. Almost all IAV gene products determine the pathogenesis of IAV in a host [7]. However, the HA, viral receptor-binding protein; NS1, the main virulence factor which antagonizes the host’s innate immune response; and PA, PB1, and PB2, the RNA-dependent RNA polymerase subunits, are critical for IAV pathogenesis [7]. In addition, accessory protein PA-X is critical for the shutoff of host protein synthesis, prioritizing viral protein synthesis, as well as downregulating the expression of host innate immune response proteins [26]. Similarly, several host proteins, promoting or restricting viral entry, viral RNA replication, transcription and translation, and viral assembly and release, play important roles in IAV pathogenesis [27,28]. In addition, host conditions like age, co-morbidities, and immune strength also play an integral role in IAV disease progression, duration, and severity [25,28]. 

During the early stages of infection, the host sensing of an invading virus particle and subsequent activation of the host’s innate antiviral response are critical in detecting and preventing the progress of a virus infection. IAV infection is recognized by one of the host cell pattern recognition receptors (PRRs)—Toll-like receptors (TLRs), retinoic acid-inducible gene I (RIG-I)-like receptors (RLRs), nucleotide-binding oligomerization domain (NOD)-like receptors (NLRs), and newly discovered Z-DNA binding protein 1 (ZBP1)—in a cellular compartment [29]. Such recognition prompts a coordinated two-pronged host innate response—interferon-mediated antiviral response and interleukin 1 beta-mediated inflammatory response—which control and clear the IAV infection [29]. This innate antiviral system comprises a vast repertoire of proteins, e.g., transcription factors and effectors, interleukins and associated effectors, interferons and interferon-stimulated genes, and is an intricate network of proteome [30]. In general, multiple factors, such as alternative splicing, alternate translation start, protein assembly and protein modifications, contribute to the complexity of the proteome [31]. In particular, protein modifications are critical for cellular response to stress, and virus infection is a form of cellular stress. Phosphorylation, one of the first protein modifications to be discovered [32], and ubiquitination have been identified and extensively studied as being central to the host’s innate antiviral response to virus infections, including of IAV [33]. Now, over 300 protein modifications are known to occur on over 250,000 sites in the human proteome [34]. However, the regulatory roles of many of these protein modifications and their crosstalk with each other are yet to be fully understood in human physiology, let alone in virus infections, including IAV. One such protein modification is acetylation.

## 3. Acetylation

Acetylation is the transfer of an acetyl group to an amino, thiol, or hydroxyl group of a small molecule or a protein [35]. On proteins, acetylation is known to occur on lysine, serine, threonine, or histidine side chains [35] or at the N-terminus [36]. Therefore, acetylation is known to exist in three types: lysine acetylation, N-terminal acetylation, and O-acetylation [37]. Lysine acetylation and N-terminal acetylation are known to be common in humans and occur post-translationally [38] and co-translationally [36], respectively. But, N-terminal acetylation can also occur post-translationally [39]. Lysine acetylation and N-terminal acetylation are catalyzed by histone acetyltransferases (HATs)—also known as lysine acetyltransferases (KATs) and N-terminal acetyltransferases (NATs), respectively. Furthermore, lysine acetylation is reversible and can be removed by histone deacetylases (HDACs)—also known as lysine deacetylases (KDACs). However, N-terminal acetylation is believed to be irreversible, and an “N-terminal deacetylase” has yet to be discovered [36]. 

Acetylation was initially discovered to be an epigenetic modification on histones [40]. Soon after, the acetylation (and methylation) of histones was discovered to play a role in gene expression [41]. These findings kicked off a cascade of further discoveries where (1) deacetylation inhibitors and the acetylation of important proteins like tubulin were discovered; (2) new HDACs or HATs and their functions were identified, and their structures were solved; and (3) new proteomic screening platforms were established to identify acetylation sites [38]. The significance of acetylation during gene expression has been studied extensively [42,43]. However, acetylation is now known to be ubiquitous, occurs on both histone and non-histone proteins, and is known to play a critical role in diverse cellular processes [38,44,45]. As a modification, acetylation influences protein stability and folding, protein–protein interactions, and protein trafficking and subcellular localization; hence, it is critical in cellular metabolism and cellular response to stress [36,38,39,46]. Any imbalance in acetylation via aberrant activity or expression of the HATs, NATs, or HDACs in humans contributes to various cancers and cardiovascular, inflammatory and infectious diseases, and developmental, neurodegenerative, and genetic disorders [47,48]. Consequently, acetylation can be a therapeutic target for treating such diseases and disorders, particularly different types of cancers [38,49]. This would also be the case for viral diseases because viruses, including IAV, exploit the host cell machinery to multiply.

## 4. Acetylation and Influenza A Virus

The first indication of the role of acetylation in IAV infection came to light in 2007, when it was discovered that the level of acetylated tubulin is increased in epithelial cells infected with the IAV. This occurred as a consequence of the downregulation of the activity of tubulin deacetylase, HDAC6 [50,51]. Furthermore, an additional increase in the acetylation level due to treatment with an HDAC inhibitor, trichostatin A, enhanced IAV replication in epithelial cells [50]. This indicated that acetylation is favourable to IAV infection, and further investigations into the role of individual HDACs and HATs/NATs during IAV infection could reveal novel determinants of IAV pathogenesis. Indeed, since then, consistent with the above initial findings, several HDACs [52,53,54,55,56,57,58,59,60,61,62,63,64] and HATs/NATs [65,66,67,68] have been identified as anti-IAV factors and pro-IAV factors, respectively.

### 4.1. HDACs Are Anti-IAV Factors

So far, there are 18 human HDACs known. Based on the sequence similarity, HDACs have been classified into four classes—I, II, III, and IV (Table 1). Class I consists of HDAC1, HDAC2, HDAC3, and HDAC8. Class II has been further divided into classes IIa and IIb. The former consists of HDAC4, HDAC5, HDAC7, and HDAC9, and the latter consists of HDAC6 and HDAC10 (both are structurally related and possess two deacetylase domains). Class III has seven members, which are known as sirtuins: SIRT1, SIRT2, SIRT3, SIRT4, SIRT5, SIRT6, and SIRT7. Finally, class IV has only one member: HDAC11 [47,48]. A potential antiviral role for members of all four HDAC classes has been investigated in IAV infection by using the cell culture and mouse models and employing experimental strategies like treatment with specific HDAC inhibitors or protein and knockdown, knockout, and the overexpression of individual HDAC genes. All members of class I [52,53,54,55]; three members of class II—HDAC4 [56], HDAC6 [57,58,59,60,61], and HDAC10 (Henzeler et al., unpublished); all members of class III [62,63]; and the sole member of class IV, HDAC11 [64] have been found to exhibit anti-IAV properties (Table 1). In cells deficient with the activity or expression of an HDAC, the growth of IAV was increased [53,54,55,56,57,62,64], and in mice lacking the expression of an HDAC, IAV-induced morbidity or mortality was increased [52,61]. In contrast, IAV growth was decreased in cells overexpressing an HDAC [55,56,57,64], and IAV-induced morbidity or mortality was decreased in mice overexpressing an HDAC or treated with a recombinant HDAC protein [58,59].

### 4.2. HATs/NATs Are Pro-IAV Factors

Multiple human HATs/NATs are known, and most of them are classified in three families (Table 2)—the p300/CBP (cAMP response element-binding protein (CREB)-binding protein) family, the GNAT (general control non-depressible 5 (Gcn5)-related N-acetyltransferase) family, and the MYST (monocytic leukemia zinc finger protein (MOZ), Ybf2/Sas3, Sas2, Tip60) family [69]. The p300/CBP family comprises two members—p300 and CBP; the GNAT family possesses seven members—HAT1, Gcn5/KAT2A, P300/CBP-associated factor (PCAF)/KAT2B, Elongator complex protein 3 (ELP3), ATase1/NAT8, ATase2/NAT8B, and Gcn5L1/Biogenesis of lysosome-related organelles complex 1 subunit 1 (BLOC1S1); and the MYST family contains five members—KAT5/TIP60/PLIP, KAT6A/MOZ/MYST3, KAT6B/MORF/MYST4, KAT7/HBO1/MYST2, and KAT8/MOF/MYST1 [69]. The NATs are a subfamily of GNATs and are known to comprise eight types—NatA, NatB, NatC, NatD, NatE, NatF (NAA60), NatG, and NatH (NAT6/NAA80/FUS2) (Table 2). Each NAT is composed of a catalytic subunit comprising an N-alpha-acetyltransferase (NAA) and an auxiliary subunit comprising Huntingtin-Interacting Protein K (HYPK) and another NAA [70,71]. The HATs/NATs, NAT9/EBS, ESCO1, and ESCO2 have yet to be classified [69]. Consistent with the role of HDACs, some HATs/NATs have been described to exhibit pro-IAV properties (Table 2). IAV infection was inhibited in cells in which the expression of Gcn5 [65], PCAF [65], NatB [67], and NatF (also known as NAA60) [68] was either knocked out or down. Furthermore, an inhibitor of p300 and CBP inhibited IAV infection both in the cell culture and animal model (mice) [66].

## 5. Proviral Mechanisms of Acetylation during Influenza A Virus Infection

Three mechanisms have emerged so far through which acetylation promotes IAV replication: (1) by reducing the host’s sensing of incoming IAV particles, (2) by dampening the host’s innate antiviral response against IAV, and (3) by increasing the stability and functionality of IAV proteins. RIG-I is a critical component of RLRs and is one of the host PRRs, which detect infection by binding the incoming viral RNAs and inducing the downstream cascade of innate antiviral response to contain infection. It has been discovered that RIG-I is acetylated at lysine 858 and 909 in uninfected or resting cells [72]. Such acetylation of RIG-I prevents its binding to viral RNA and subsequent detection of IAV infection and the induction of innate antiviral response cascade [60,72]. Likewise, enhanced acetylation downregulates the expression of type I interferons (interferon alpha and beta) and cytokines (CXCL10 and interleukin 6 and 15) as well as interferon-stimulated genes (ISGs) such as IFITM3, ISG15, ISG20, and viperin in IAV-infected cells [52,54,55,56,60,61,64,65,68,73]. 

Furthermore, most of the IAV proteins have been identified to be either lysine-, serine- and/or N-terminally acetylated (Table 3). The M1 is acetylated at lysine 95 [74] and M2 at lysine 60 [75], whereas NP is acetylated at multiple lysine residues viz. 31, 77, 90, 113, 184, 229, and 325 [74,76,77]. The NS1 is acetylated at lysine 108 [78], and PA is acetylated at lysine 19, 104 and 664 [74,79,80]. Also, the M1, NP, and PA are acetylated at serine residues: M1 at serine 195, 196, and 207; NP at serine 274, 283, 287, 326, and 403; and PA at serine 631 [74]. Finally, NS1, NS2 (NEP), PB1 [74], and PA-X [67] are acetylated at the N-terminus (Table 3). The acetylation of IAV proteins promotes their stability [80] and function [67,76,77,78,79,81] and, consequently, IAV replication [67,77,78]. Specifically, lysine acetylation promotes the interferon-antagonizing function of NS1 [78] and stability [80], as well as the endonuclease activity [79] of PA. Furthermore, N-terminal acetylation promotes the nuclear import as well as host shutoff activity of PA-X [67,81]. In addition, many host proteins, e.g., actin, eIF4GI, exportin, importin, mTOR (mammalian target of rapamycin), NHP2L1, nucleophosmin, SF3B2, and tubulin, are also hyper-acetylated at the lysine or serine residues or at the N-terminus in IAV-infected cells [50,55,74]. Many of those proteins promote viral functions during different stages of IAV infection, for example, viral RNA synthesis and processing (nucleophosmin, SF3B2, and NHP2L1), viral protein synthesis (mTOR and eIF4GI), viral protein trafficking via nucleus and cytoplasm (importin, exportin, and tubulin), and viral budding (actin) [57,74].

## 6. Antagonism of HDACs during Influenza A Virus Infection

HDACs, as the “erasers of acetylation”, interfere with the pro-IAV function of acetylated viral proteins [80] and host proteins [57,60,73] by deacetylating them. Hence, IAV must antagonize the HDACs to maintain optimal acetylation levels of those proteins and successfully replicate. IAV has been discovered to antagonize the HDACs by downregulating both their expression [53,54,55,56,64,82,83] and activity [50,55,57] in infected cells. To downregulate the expression of HDACs at the mRNA level, IAV employs both viral and host components. For example, IAV employs its proteins, PA-X and PA, to downregulate the mRNA levels of HDAC4 [56] and HDAC6 [82], respectively. Notably, both PA-X and PA possess the endonuclease function, and particularly, PA-X is instrumental in host shutoff activity [26]. Furthermore, IAV employs a micro-RNA, miR-21-3p, to downregulate the level of HDAC8 mRNA [53]. To downregulate the expression of HDACs at the protein level, IAV employs host proteasome- and caspase-mediated protein degradation mechanisms, which seem to be tailored for the HDACs from a particular class. The class I HDACs, HDAC1 and HDAC2, are degraded in infected cells by proteasome pathway [54,55], whereas, the class II HDACs, HDAC4 and HDAC6, are degraded by caspases [56,82].

## 7. Prospects

Like other viruses, IAV is an obligate intracellular parasite, and hence exploits and antagonizes the host machinery to multiply and cause disease, respectively. Protein modifications like acetylation, which drive the complexity of the host proteome, are bound to play an integral role in IAV multiplication and pathogenesis. The literature generated in this space over the last 15 years indicates that this is the case, and acetylation plays a pro-IAV role and is one of the determinants of IAV pathogenesis. However, we are a long way away from elucidating the significance of acetylation and other epigenetic modifications like methylation during the infection of type A influenza viruses. For the other three influenza virus types (B, C, and D), there is a paucity of literature in this space. Nevertheless, with further refinements in the existing genomics and proteomics platforms and gene editing techniques, it will be easier and quicker to generate knowledge regarding the extent and significance of the involvement of acetylation in influenza virus infections. Subsequently, such knowledge could contribute to developing alternative influenza virus intervention strategies as well as understanding the molecular bases of flu disease severity in different ethnic populations.

## Figures and Tables

**Table 1:** The HDACs with known anti-IAV functions.

Class	HDACs	HDACs Exhibiting anti-IAV Function
I	HDAC1, HDAC2, HDAC3, HDAC8	HDAC1 [55], HDAC2 [54], HDAC3 [52], HDAC8 [53]
IIa	HDAC4, HDAC5, HDAC7, HDAC9	HDAC4 [56]
IIb	HDAC6 ^1^, HDAC10 ^1^	HDAC6 [57,58,59,60,61]
III	SIRT1, SIRT2, SIRT3, SIRT4, SIRT5, SIRT6, SIRT7	SIRT 1-7 [62,63]
IV	HDAC11	HDAC11 [64]

^1^ Contains two deacetylase domains.

**Table 2:** The HATs/NATs with known pro-IAV functions.

Family	HATs/NATs	HATs/NATs Exhibiting Pro-IAV Functions
p300/CBP	p300, CBP	p300, CBP [66]
GNAT	HAT1, Gcn5/KAT2A, PCAF/KAT2B, ELP3, ATase1/NAT8, ATase2/NAT8B, GCN5L1/BLOC1S1 and NATs subfamily—NatA, NatB, NatC, NatD, NatE, NatF (NAA60), NatG, NatH (NAT6/NAA80/FUS2)	Gcn5/KAT2A, PCAF/KAT2B [65], NatB [67], NatF (NAA60) [68]
MYST	KAT5/TIP60/PLIP, KAT6A/MOZ/MYST3, KAT6B/MORF/MYST4, KAT7/HBO1/MYST2, KAT8/MOF/MYST1	
Unknown ^1^	NAT9/EBS, ESCO1, and ESCO2 ^1^	

^1^ Yet to be classified [69].

**Table 3:** Acetylation on IAV proteins.

Acetylation Type	IAV Protein (*Acetylation Position*)
Lysine (K)	M1 (*K95*) [74], M2 (*K60*) [75], NP (*K31*, *K77*, *K90*, *K113*, *K184*, *K229*, *K325*) [74,76,77], NS1 (*K108*) [78], PA (*K19*, *K104*, *K664*) [74,79,80]
Serine (S)	M1 (*S195*, *S196*, *S207*), NP (*S274*, *S283*, *S287*, *S326*, *S403*), PA (*S631*) [74]
N-terminus	NS1, NS2 (NEP), PB1 [74], PA-X [67]

## Data Availability

Not applicable.
